# Distinct molecular profiles associated with early vs late genome instability in cancer

**DOI:** 10.1038/s41598-026-44528-y

**Published:** 2026-04-06

**Authors:** Lucie Gourmet, James Lam, Adam Pennycuick, Luis Zapata, Parag Mallick, Simon Walker-Samuel

**Affiliations:** 1 Centre for Computational Medicine, University College London, London, UK; 2 Lungs for Living Research Centre, UCL Respiratory University College London, London, UK; 3 Centre for Evolution and Cancer Institute of Cancer Research, London, UK; 4 Department of Radiology, Stanford University, Palo Alto, California USA

**Keywords:** Cancer, Computational biology and bioinformatics, Genetics, Oncology

## Abstract

The temporal order in which cancer hallmark phenotypes emerge can influence tumor behavior and prognosis. In prior work, we used variant allele frequency (VAF) analysis to infer hallmark acquisition order in over 30,000 tumors, identifying two dominant evolutionary trajectories: one characterized by early genome instability (EGI) and the other by late genome instability (LGI). These trajectories were associated with distinct survival outcomes. Here, we investigate the molecular and microenvironmental features that differentiate these two groups, using data from The Cancer Genome Atlas (TCGA). We show that EGI tumors exhibit higher aneuploidy and are enriched for mutational signatures linked to homologous recombination deficiency, particularly SBS3, while LGI tumors show greater prevalence of environmentally associated signatures, including those linked to UV exposure and haloalkanes. Immune profiling revealed that activated mast cells, dendritic cells, and NK cells are enriched in EGI tumors, whereas resting mast cells are strongly associated with LGI tumors. Stemness scores were also significantly higher in EGI tumors, while epithelial-mesenchymal transition (EMT) metrics did not differ between groups. These findings suggest that the timing of genome instability is associated with distinct mutational processes, immune states, and plasticity features. Understanding these differences may provide insight into tumor evolution and offer opportunities for stratified therapeutic intervention based on hallmark ordering.

**Supplementary Information:**

The online version contains supplementary material available at 10.1038/s41598-026-44528-y.

## Introduction

The progression of cancer is driven by the sequential acquisition of biological capabilities, known as the cancer hallmarks^1–3^, that enable malignant growth and survival. Although all cancer hallmarks are typically present by the time of diagnosis^4^, the sequence in which they are acquired may vary across tumors and influence their behaviour and therapeutic response.

In prior work, we used variant allele frequency (VAF) and the ratio of non-synonymous to synonymous substitution rates (dN/dS) to determine the relative ordering of mutations affecting the ten canonical cancer hallmarks across over 30,000 tumors^5^. This study revealed two major patient trajectories: one in which genome instability appeared early and was often associated with TP53 mutations, and one where it occurred late. Tumors exhibiting early genome instability (EGI) were more likely to harbor TP53 mutations, displayed distinct transcriptional profiles, and were associated with poorer patient survival compared to those with late genome instability (LGI). These findings suggested that hallmark timing, particularly of genome instability, may reflect underlying differences in tumor evolution and biology.

The present study builds on this observation by investigating the molecular and microenvironmental features that distinguish EGI and LGI tumors. Because tumors with early genome instability (EGI) were associated with a worse prognosis compared to those with late genome instability (LGI), we aimed to identify the molecular mechanisms underlying this clinical difference. Specifically, we examine the patterns of mutational signatures (both single base substitutions and copy number alterations) along with measures of immune infiltration, stemness, and epithelial-mesenchymal transition (EMT). Our aim, using data from The Cancer Genome Atlas (TCGA), is to determine whether tumors with different timing of genome instability show systematic differences in their mutational processes and tumor microenvironments. Such differences could shed light on the selective pressures that shape hallmark acquisition and may help guide the development of stratified therapeutic approaches based on hallmark dynamics.

## Results

**Fig. 1:**
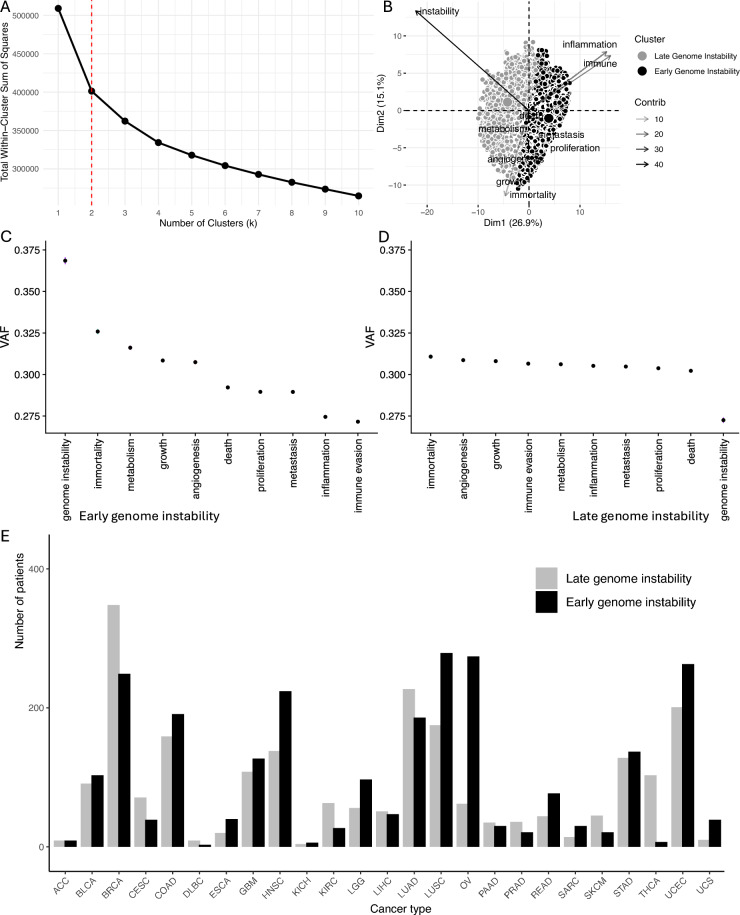
Clustering validation and characterization of cancer hallmark trajectories. (**A**) Elbow plot showing total within-cluster sum of squares for k=1–10 clusters. Red dashed line indicates optimal k=2. (**B**) PCA biplot of patient samples colored by cluster assignment. Arrows represent cancer hallmark loadings (contribution to principal components). (**C**-**D**) Variance allele frequency (VAF) of cancer hallmarks ordered by rank in (**C**) Early Genome Instability (EGI) tumors, where genome instability is acquired first. (**D**). Late Genome Instability (LGI) tumors, where genome instability is acquired late. Error bars represent standard error. (**E**) Histogram showing the distribution of early and late genome instability clusters within each cancer type.

We first investigated the association between genetic/immune features in patients with early vs late genome instability. We used k-means clustering to determine patients with early genome instability and late genome instability (Fig 1) and hypothesized that patients with increased structural genomic instability, would have distinctive copy number signatures. The two clusters exhibited significantly different VAF distributions (Wilcoxon rank sum test, p < 2.2e-16). We performed Mann-Whitney U tests comparing VAF distributions between clusters for each cancer hallmark, with Benjamini-Hochberg correction for multiple testing. All hallmarks showed highly significant differences (adjusted p < 10e-6), indicating robust biological distinctions. The largest effects were observed for immune evasion (median VAF difference = 0.049, p < 10e-178) and inflammation (difference = 0.047, p < 10e-181). Notably, late genome instability tumors show higher VAF for immune/inflammation hallmarks, suggesting these acquire immune-related alterations as early founder events, while early genome instability tumors show the opposite pattern, with genome instability occurring earlier. The consistency across hallmarks and sample sizes indicates these represent biologically meaningful differences in tumor evolutionary trajectories. We used logistic regression to identify associations between immune cell populations and genome instability timing. There were 3142 samples in early genome instability samples and 2914 samples in late genome instability samples.

### Early genome instability has increased mutation burden and aneuploidy

**Fig. 2:**
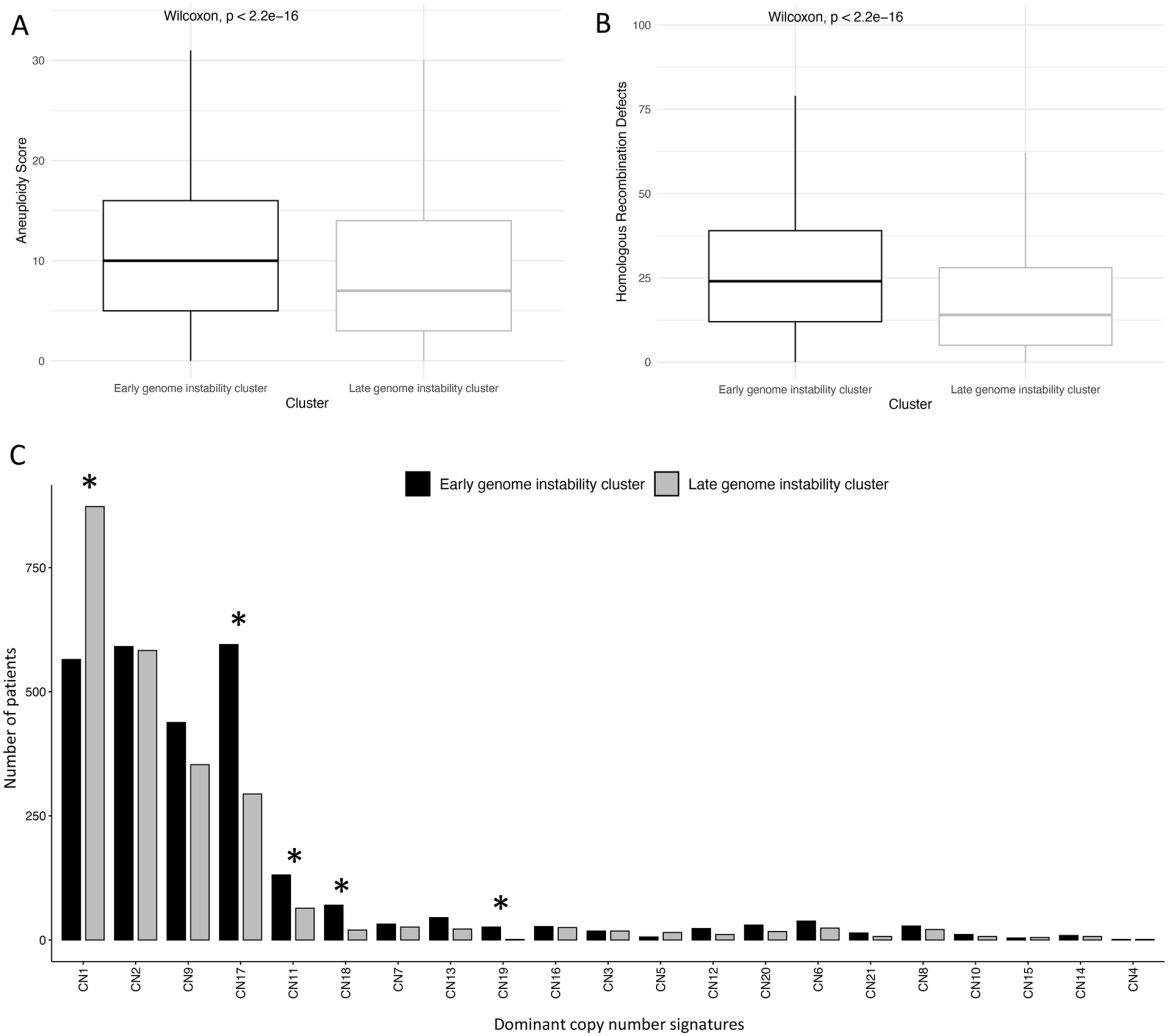
The role of mutational burden in hallmark clustering. (**A**) Boxplot of the aneuploidy score. (**B**) Boxplot for homologous recombination defects. (**C**) Histogram showing the number of dominant copy number signatures per patient per cluster. Asterisks denote statistical significance with a p value adjusted for multiple comparison <0.05.

We hypothesized that patients with early genome instability (EGI-tumours), meaning being in the cluster with genome instability ranked first, would exhibit a higher mutational burden. We found that EGI-tumours always displayed a significantly higher aneuploidy score compared to the late genome instability patients (LGI-tumours) (Wilcoxon, p< 2.2 e-16, Fig2A), homologous recombination defects (Wilcoxon, p< 2.2 e-16, Fig 2B), silent mutation rate (Supp Fig 1) and nonsilent mutation rate (Supp Fig 2). These results were consistent with our hypothesis, and we investigated the number of dominant signatures across patients. For each copy number signature, we reported the number of patients having it as a dominant signature (Fig 2C). We found that CN1, primarily a signature of a diploid genome, exhibited a significantly higher number of LGI-tumours. (Fisher’s exact test adjusted p value: 2.345e-33). CN1 is also negatively associated with many signals of a perturbed genome such as APOBEC SBS signatures (SBS2, SBS13) and TP53 mutations. The lower prevalence of CN1 in EGI-tumours aligns with its characteristic early genome instability, typically initiated by TP53 mutations leading to increased aneuploidy. Moreover, CN17 and CN11 was associated with a greater number of EGI-tumours (Fisher’s exact test adjusted p value: 8.880e-19 and 3.068e-04 respectively). CN17 is a signature of homologous recombination deficiency while CN11 is indicative of loss events before two genome doubling events (loss-of-heterozygosity). Two other signatures (CN18 and CN19) were associated with a greater number of EGI-tumours but their aetiologies are unknown (Fisher’s exact test adjusted p value: 2.424e-05 and 8.041e-05 respectively). Bootstrap analysis revealed significant associations between CN1 and LGI (adjusted p value: 2.345e-33) and between CN17 and EGI (adjusted p value: 8.880e-19) (Supp Fig 3). Thus, the EGI-tumours and LGI-tumours display different numbers of copy number signatures.

### Late genome instability is associated with a greater number of environmental signatures

**Fig. 3:**
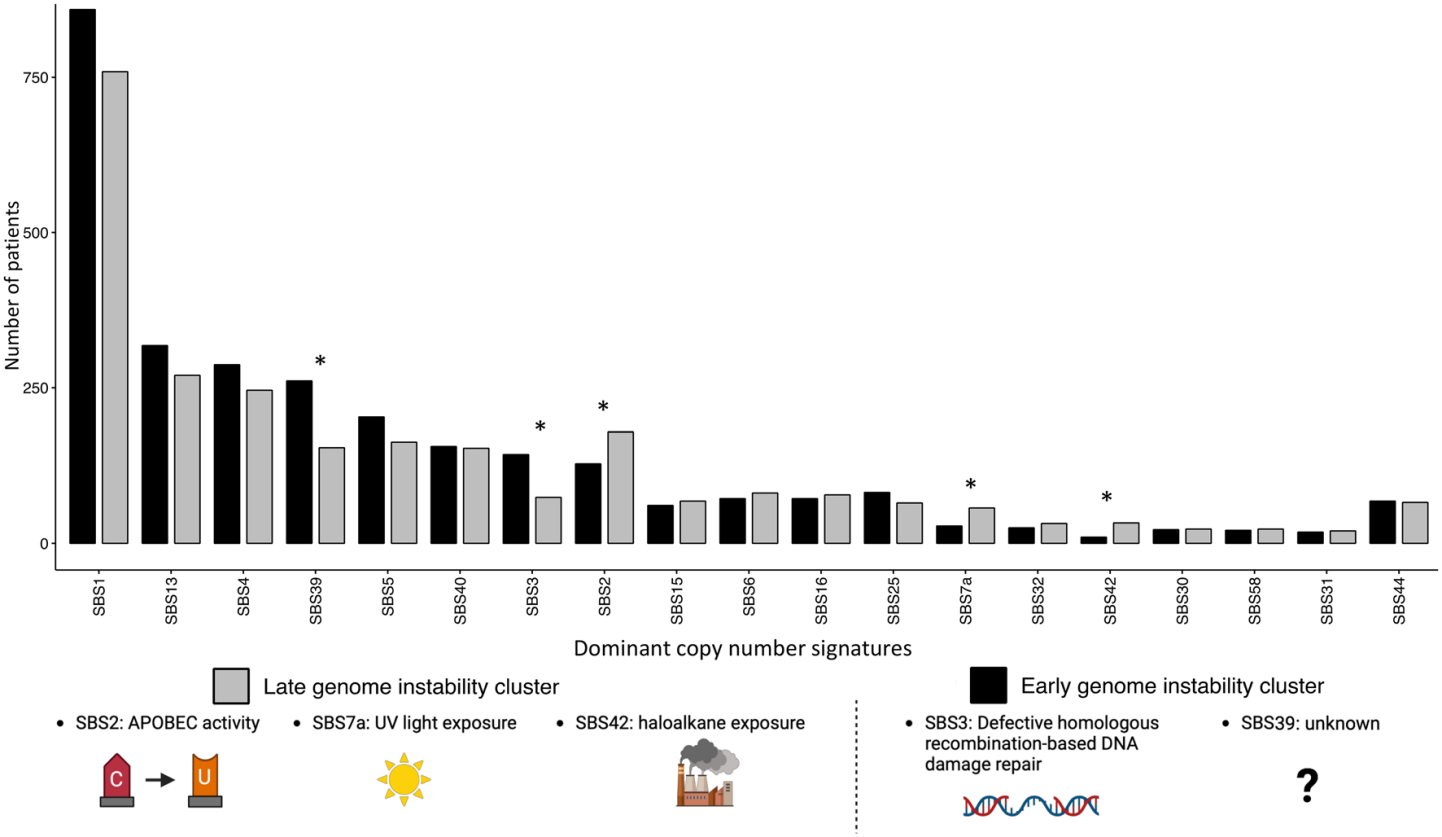
Histogram showing the number of dominant single base substitution mutational signatures in each patient of each cluster. Asterisks denote statistical significance with a p value adjusted for multiple comparison <0.05.

When investigating SBS mutational signatures, we hypothesized that LGI tumours would be more influenced by exogenous mutational processes than EGI tumours as the later onset of instability allows more time for environmental interactions. We found 5 SBS signatures with significant differences in the number of EGI and LGI-tumours (Figure 3). EGI-patients had a higher number of patients with dominant signatures SBS39 (Fisher’s exact test adjusted p value: 2.34e-04), of unknown origin, and SBS3, associated with defective homologous recombination-based DNA repair (Fisher’s exact test adjusted p value: 9.37 e-04). Conversely, LGI-patients were associated with SBS2 (APOBEC activity, enzymes that convert cytidine to uracil), SBS7a (UV light) and SBS42 (haloalkane exposure) (Kruskal-Wallis adjusted p value: 3.713e-03, 6.421e-3, 3.713e-03). Unexpectedly, SBS2 showed a stronger association with LGI-patients, despite this cluster’s previous link to CN1, which is negatively associated with APOBEC signatures. However, the number of LGI-patients associated with CN1 (n = 873) was substantially higher than those associated with SBS2 (n = 179). The other signatures SBS7a and SBS42 were due to environmental exposure, supporting our hypothesis that LGI-patients are more influenced by external pressures. The most common signature in both clusters was SBS1, which results from the spontaneous or enzymatic deamination of 5-methylcytosine to thymine. SBS1 is known to correlate with age. When performing the bootstrap analysis to validate the difference of mutational signatures between EGI and LGI-tumours, we found that SBS2, SBS7a and SBS3 had a significant p value and a large effect size (Supp Fig 4). Furthermore, we found that no significant correlation between the number of substitutions per gene and the mean VAF per gene, indicating that substitution signature differences between VAF-based clusters are not artifacts of the clustering approach (Supp Fig 5).

### The state of mast cells impacts both early and late genome instability

**Fig. 4:**
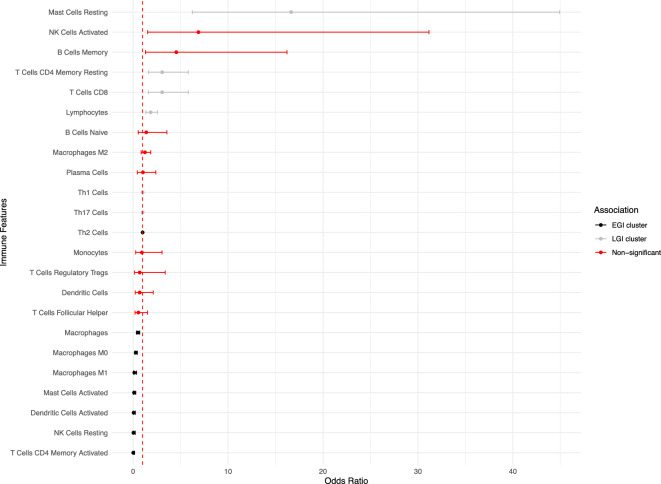
Forest plot showing odds ratios (OR) and 95% confidence intervals for the association between immune cells with the LGI cluster compared to the EGI cluster. The vertical dashed red line at OR = 1 represents no association. Features are color-coded by Bonferroni-corrected statistical significance and direction: black indicates significant association with EGI cluster (Bonferroni-adjusted p < 0.05, OR < 1); grey indicates significant association with LGI cluster (Bonferroni-adjusted p < 0.05, OR > 1); red indicates non-significant associations after multiple testing correction (Bonferroni-adjusted p ≥ 0.05).

Given our observation that EGI tumours are associated with a greater mutational burden, we next sought to test the hypothesis that this would be accompanied by differential immune cell enrichment. Using odds ratio analysis with Bonferroni correction for multiple testing, we assessed the association between immune cell abundances and early or late-onset genome instability (Fig 4).

Resting mast cells were strongly associated with LGI tumours (OR=16.6, 95% CI: 6.24–44.9; Bonferroni-adjusted p=5.25e-7), while activated mast cells were significantly enriched in EGI tumours (OR=0.0960, 95% CI: 0.0333–0.273; Bonferroni-adjusted p=2.81e-4). Several other immune cell types demonstrated significant associations with EGI tumours after multiple testing correction, including activated dendritic cells (OR=0.0548, 95% CI: 0.0128–0.229; Bonferroni-adjusted p=1.82e-3), resting NK cells (OR=0.0311, 95% CI: 0.00435–0.219; Bonferroni-adjusted p=1.18e-2), CD4 activated memory T cells (OR=0.0115, 95% CI: 0.000837–0.152; Bonferroni-adjusted p=1.72e-2), and macrophages including M0 (OR=0.274; Bonferroni-adjusted p=3.30e-6), M1 (OR=0.123; Bonferroni-adjusted p=4.37e-3), and overall macrophage populations (OR=0.499; Bonferroni-adjusted p=1.38e-3). This activation pattern in EGI tumours aligns with their early genome instability profile, potentially allowing for prolonged tumour-immune cell interactions.

Conversely, LGI tumours showed significant enrichment of CD8 T cells (OR=3.05, 95% CI: 1.61–5.82; Bonferroni-adjusted p=1.57e-2), CD4 memory resting T cells (OR=3.06, 95% CI: 1.61–5.81; Bonferroni-adjusted p=1.43e-2), and lymphocytes (OR=1.85, 95% CI: 1.33–2.56; Bonferroni-adjusted p=5.08e-3). Several immune cell populations showed no significant association with either cluster after correction for multiple testing, including activated natural killer cells, B cell subsets (naive and memory), M2 macrophages, monocytes, plasma cells, follicular helper T cells, regulatory T cells, and general dendritic cell populations. To further elucidate the immunological landscape, we investigated the impact of our clustering on broader immune and stemness features.

### Stemness metrics are associated with early genome instability

**Fig. 5:**
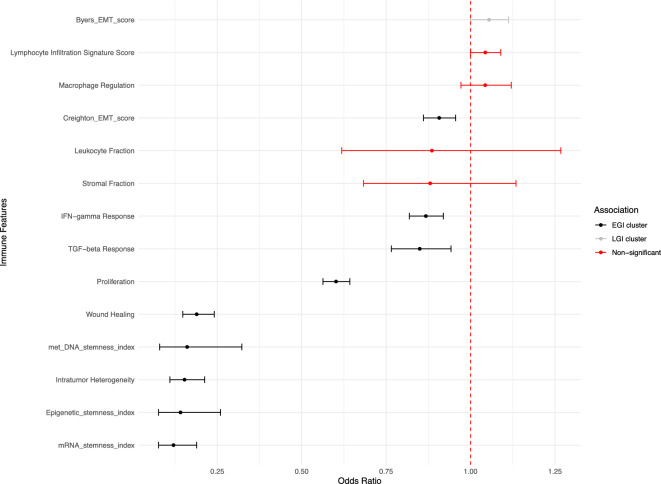
Forest plot showing odds ratios (OR) and 95% confidence intervals for the association between immune features with the LGI cluster compared to the EGI cluster. The vertical dashed red line at OR = 1 represents no association. Features are color-coded by Bonferroni-corrected statistical significance and direction: black indicates significant association with EGI cluster (Bonferroni-adjusted p < 0.05, OR < 1); grey indicates significant association with LGI cluster (Bonferroni-adjusted p < 0.05, OR > 1); red indicates non-significant associations after multiple testing correction (Bonferroni-adjusted p ≥ 0.05).

We next investigated epithelial-mesenchymal transition (EMT) and stemness metrics, hypothesizing that these would be more strongly associated with EGI-tumours. Interestingly, the two EMT scores showed divergent patterns (Fig 5): the Creighton EMT score was significantly associated with EGI-tumours (OR = 0.907, 95% CI: 0.861–0.956; Bonferroni-adjusted p = 3.75e-3), while the Byers EMT score showed a weak association with LGI-tumours (OR = 1.05, 95% CI: 1.00–1.11; Bonferroni-adjusted p = 0.70), though this latter association did not survive multiple testing correction. In contrast, all stemness indices showed robust associations with EGI-tumours: mRNA stemness index (OR = 0.120, 95% CI: 0.0765–0.189; Bonferroni-adjusted p = 7.16e-19), methylated DNA stemness index (OR = 0.161, 95% CI: 0.0794–0.323; Bonferroni-adjusted p = 4.52e-6), and epigenetic stemness index (OR = 0.141, 95% CI: 0.0763–0.260; Bonferroni-adjusted p = 5.12e-9). This strong association suggests that early genomic instability events in this cluster may preferentially occur in stem-like cell populations, potentially driving the observed tumour phenotypes.

Consistent with the early genome instability characteristic of this cluster, intratumour heterogeneity (OR = 0.153, 95% CI: 0.110–0.213; Bonferroni-adjusted p = 1.35e-27) and wound healing signatures (OR = 0.189, 95% CI: 0.148–0.241; Bonferroni-adjusted p = 1.63e-39) were both strongly associated with EGI-tumours. Unexpectedly, proliferation was also significantly associated with EGI-tumours (OR = 0.602, 95% CI: 0.563–0.643; Bonferroni-adjusted p = 8.00e-50), suggesting that despite early genomic instability, these tumours maintain active proliferative capacity. Additionally, both IFN-gamma response (OR = 0.868, 95% CI: 0.819–0.919; Bonferroni-adjusted p = 2.23e-5) and TGF-beta response (OR = 0.850, 95% CI: 0.766–0.942; Bonferroni-adjusted p = 2.88e-2) showed significant associations with EGI-tumours, indicating active immune signalling in this cluster.

The remaining parameters (stromal fraction, leukocyte fraction, macrophage regulation, and lymphocyte infiltration) showed no significant associations with either cluster after correction for multiple testing.

Both EGI and LGI clusters are represented across a broad range of tumor types, although their relative proportions vary by cancer type (Fig. 1E). This indicates that the observed EGI/LGI dichotomy is not driven by a single tumor entity, while also highlighting that cancer type composition may contribute to some of the observed pan-cancer differences. A systematic evaluation of cluster-associated features within individual cancer types is left for future studies.

## Discussion

In this study, we investigated the mutational and microenvironmental features associated with the timing of genome instability in cancer, focusing on tumors that acquire this hallmark either early or late in their evolutionary trajectory. Building on our prior analysis of over 10,000 tumors, which revealed two major trajectories distinguished by the relative timing of genome instability (5), we sought to understand the molecular correlates that might underlie these divergent paths. Our results show that early genome instability (EGI) tumors are characterised by higher levels of aneuploidy, enrichment for specific mutational signatures such as SBS3 and CN17, increased stemness, and distinct immune cell profiles compared to late genome instability (LGI) tumors.

Immune profiling further revealed differences between the two groups. EGI tumors were associated with higher levels of activated mast cells, activated dendritic cells, and activated NK cells, while LGI tumors showed a strong association with resting mast cells, CD8 T cells, CD4 memory resting T cells, lymphocytes. This immune contexture, together with increased stemness scores observed in EGI tumors, may reflect a more immune-evasive, plastic phenotype. One possibility is that genome instability occurring early in tumor evolution exposes neoantigens that trigger immune recognition, thereby selecting for immune-evasive strategies such as dedifferentiation and suppression of immune effector cells. The elevated stemness scores support this view, suggesting a shift toward cell states that are less immunogenic and more resistant to standard therapies.

Interestingly, while the Creighton EMT score was significantly associated with EGI-tumours, the Byers EMT score showed a weak association with LGI-tumours. Understanding EMT (epithelial-mesenchymal transition) and the TME in cancer evolution is crucial because EMT is linked to immune cell infiltration and activation of immune checkpoint pathways, which play roles in cancer survival and treatment response^6^. Interestingly, negative associations between stemness and EMT gene signatures have been reported, though stemness also influences clinical outcomes^7^.

These findings have several implications. Firstly, they suggest that the timing of genome instability is not merely a passive consequence of tumor evolution but may reflect distinct underlying biological programs. Secondly, the association of EGI tumors with HRD signatures raises the possibility that these tumors may be more sensitive to DNA-damaging agents or PARP inhibitors, although their immune-evasive nature and high stemness could counteract this sensitivity. Thirdly, the presence of specific immune cell patterns suggests that immune contexture is shaped in part by the evolutionary timing of key hallmarks and may itself influence subsequent tumor behaviour.

In our prior analysis, EGI tumors were associated with significantly poorer survival outcomes across multiple cancer types. The current study expands on this by showing that EGI tumors are also enriched for mutational signatures associated with homologous recombination deficiency and have a distinct immune landscape characterized by increased activated mast cells and higher stemness metrics. These features may help explain their aggressive behavior and suggest they may benefit from therapies targeting DNA repair or stem-like cell states.

As we also showed previously, TP53 mutations were central to early genome instability. In the current study, CN17 and CN11, both associated with TP53 loss and genome doubling, were enriched in the EGI cluster, supporting the link between TP53 dysfunction and early instability. Interestingly, LGI tumors were enriched for mutational signatures SBS7a (UV light) and SBS42 (haloalkanes), suggesting that strong environmental mutagens may delay the emergence of genome instability or mask its effects in the hallmark ordering process. This supports our earlier findings that melanoma and related cancers diverge from the canonical hallmark trajectory^5^.

This study has limitations. While we interpret the EGI and LGI classifications as reflecting temporal dynamics, these are inferred from static data using VAF-based ranking which does not consider tumor purity and copy number variations. Mutations in regions with high copy number amplification may appear to have inflated VAFs, while those in subclonal or low-purity samples may be underestimated. As a result, the temporal ordering of mutations may be skewed. Although a majority of cancer types exhibited a consistent hallmark ordering^5^, a subset (especially those influenced by strong environmental mutagens like UV) deviated significantly. While both CN and SBS signatures can arise from shared DNA repair defects (e.g., homologous recombination deficiency generating both SBS3 and specific CN signatures), formal integration is challenging due to differences in genomic scale, temporal dynamics, and analytical frameworks between these data types. Future studies employing multi-omic integration methods could reveal more detailed mechanistic connections between these mutational processes in the context of genome instability timing. Moreover, our analyses were conducted at the pan-cancer level, which may obscure cancer-type specific effects. Although our prior work demonstrated consistency in hallmark order across most tumor types, stratified analyses will be important to confirm whether these patterns hold within individual cancer contexts. We acknowledge that our separate analyses of mutational and copy-number signatures represent a limitation.

Moreover, the data used for clustering are derived from a single timepoint, which limits our ability to accurately distinguish between EGI and LGI tumours. Ideally, a longitudinal dataset with serial tumour samples collected over time would enable direct observation of when genome instability emerges during tumour evolution such as the TRACERx studie^8,9^. Tracking mutational burden across timepoints would allow us to more definitively assign the timing of genome instability events, rather than inferring them. In addition, the immune cell population data was derived from TCGA bulk RNA sequencing using CIBERSORT^10^ in a previous paper^11^. Bulk sequencing cannot fully capture the spatial distribution of immune cells within the tumor microenvironment, using single cell sequencing would have been more informative. Overall, our study’s findings are associative rather than causative, and functional validation would be needed to confirm the biological significance of the relationships between cancer hallmark ordering and mutational signatures/immune cells. Since different cancer types are known to harbour distinct mutational signatures, the observed differences in substitution patterns between clusters may be partially driven by the underlying cancer type distribution.

In conclusion, the timing of genome instability is associated with distinct mutational processes, immune features, and plasticity states. These differences may contribute to the heterogeneity of tumor behaviour and could inform strategies for therapeutic stratification. Understanding the interplay between evolutionary trajectories and tumor phenotype may offer a new dimension for precision oncology. These findings may inform future stratified treatment strategies and enhance our understanding of evolutionary dynamics in cancer.

## Methods

### Data acquisition and pre-processing

We used publicly available data from The Cancer Genome Atlas (TCGA) (https://portal.gdc.cancer.gov/) to retrieve the variant allele frequency (VAF) values for each mutation. We plotted the mean VAF and standard error of the mean for each hallmark in Figure 1. TCGA data were annotated using the annotation step from dNdScv^12^, excluding metastatic samples and duplicated data, retaining only samples from primary tumors. The TCGA pan-cancer dataset comprises 2,626,225 mutations across 9,484 patients from 32 cancer types (https://gdc.cancer.gov/resources-tcga-users/tcga-code-tables/tcga-study-abbreviations).

We obtained different data for immune cells^11^, EMT scores^6^ and stemness index from^7^. These datasets were combined with the TCGA data using TCGA ID.

### Clustering analysis

Initially, the raw dataset, which included mean VAF for 10 hallmarks from all TCGA samples, underwent pre-processing to ensure suitability for analysis. This involved checking for missing values and outliers, with the distribution of values assessed visually through boxplots and histograms using ggplot2 (version 3.4.2) in R.

Principal Component Analysis (PCA) was performed using the prcomp function in base R. We performed PCA on cancer hallmark trajectories data to reduce dimensionality, calculating all principal components but visualizing only the first two dimensions for interpretability, while utilizing the complete set of principal components for subsequent k-means clustering to identify distinct patient subgroups. To identify the optimal number of clusters for k-means, the Elbow method was used^13^. We plotted the total within-cluster sum of squares (WSS) against a range of potential cluster numbers using the fviz_nbclust function from the factorextra package in R. The ‘elbow point,’ where the decrease in WSS diminished, indicated the optimal number of clusters.

K-means clustering was then executed with the kmeans function in R, after determining the optimal number of clusters. The cluster assignments for each sample were recorded. The performance of our clustering was assessed using the silhouette method, which evaluates how similar a sample is to its own cluster relative to other clusters. We computed the silhouette scores for each sample using the silhouette function from the cluster package in R. The average silhouette width was calculated to assess the overall quality of the clustering, with higher average silhouette widths indicating better-defined clustering.

The clustering led to 3142 samples in the early genome instability cluster and 2914 samples in the late genome instability cluster. However, because of lack of information in certain cases, the numbers were different during the signature analysis:

-SBS signatures: 3130 samples in the early genome instability cluster and 2898 samples in the late genome instability cluster.
-CN signatures: 2702 samples in the early genome instability cluster and 2394 samples in the late genome instability cluster.

These differences reflect the requirement for adequate mutation or alteration counts to perform robust signature decomposition. Samples with fewer than 50 total somatic mutations were excluded, as this is the minimum required for reliable mutational signature decomposition using the deconstructSigs algorithm.

We refer to the clusters as ‘early’ and ‘late’ genome instability (EGI and LGI) based on their position in the VAF-derived hallmark ranking described in our prior study, reflecting inferred mutational timing. Although imperfect, VAF ordering correlates with timing in bulk sequencing data and has been validated using independent metrics such as cancer cell fraction (CCF) and cross-cohort consistency^5^.

## Mutational signatures

Copy number (CN) signatures are defined using a 48-channel copy number classification scheme from 9,484 tumours. CN signature identification involves nonnegative matrix factorization and clustering based on a matrix of copy number alteration patterns segmented by features like total copy number and segment size. They have been associated with specific mechanisms such as chromothripsis, a process involving clustered rearrangements associated with oscillating copy number patterns^14^. Other aetiologies include homologous recombination deficiency, loss of heterozygosity and aneuploidy.

Single base substitutions are classified based on two factors: the type of mutation and the context of the surrounding DNA. First, there are six possible changes to the DNA bases (C>A, C>G, C>T, T>A, T>C, T>G). Second, each mutation is influenced by the two neighboring bases, one on each side, which can be any of the four DNA bases. This creates 16 possible combinations of neighboring bases. When these six mutations are combined with the 16 contexts, it results in 96 categories^15^. SBS signatures are extracted using nonnegative matrix factorization of the frequencies of mutations across the 96 categories. The relative contribution of each CN and SBS signature can be quantified for individual tumors, with the signature having the highest contribution referred to as the dominant signature. This information can provide insights into the underlying mutational processes driving tumor development and progression, potentially informing therapeutic strategies and prognosis^16,17^.

For copy number signatures, we used data extracted with sigProfiler^14^. We analyzed Copy Number Variation (CNV) patterns across two patient clusters using R and the tidyverse package suite. For each patient, we identified the dominant CNV signature by selecting the CNV type with the highest value. We then calculated the frequency of each CNV type within each cluster to characterize cluster-specific CNV distribution patterns. To assess the significance of differences in CNV frequencies between clusters, we employed either chi-square tests or Fisher’s exact tests, depending on the expected cell frequencies in the contingency tables. Chi-square tests were used when all expected frequencies were ≥5, while Fisher’s exact tests were applied otherwise. To account for multiple comparisons, we applied the Benjamini-Hochberg procedure for controlling the false discovery rate, considering adjusted p-values <0.05 as statistically significant. This approach allowed us to systematically compare CNV patterns between the two patient clusters, identify statistically significant differences, and control for multiple testing issues, potentially informing further biological or clinical investigations. Results were visualized using ggplot2 to illustrate the distribution of CNV types across clusters and highlight significant differences.

For single base substitution signature data, we used the deconstructSigs^18^ (version 1.8.0) and applied it to the pancancer TCGA cohort. Then, we repeated the same steps as for copy number analysis.

### Logistic regression analysis

We conducted logistic regression analysis to examine the association between the relative abundance of immune cell populations (estimated from RNA bulk sequencing using CIBERSORT^11^) and EGI/LGI tumours. The dataset was pre-processed to create a binary cluster variable. We analysed 25 immune cell types, including T cell subsets, B cells, natural killer cells, macrophages, and dendritic cells. For each immune cell type, we fitted a separate logistic regression model with the binary cluster as the dependent variable and the immune cell population as the independent variable. We calculated odds ratios, 95% confidence intervals, and p-values for each model. To visualize the results, we created a forest plot using ggplot2, displaying the log odds ratios and their confidence intervals for each immune cell type. This approach allowed us to systematically assess and compare the relationships between various immune cell populations and the two patient clusters, providing insights into the immunological differences between these groups.

### Bootstrap analysis

To assess the performance of copy number (CN) signature distributions between two clusters, we employed a bootstrap analysis approach. The analysis was conducted with the following packages: boot, dplyr, tidyr, ggplot2, kSamples, and effsize. We performed 1000 bootstrap replicates (R = 1000) using the boot function from the boot package. The bootstrap statistic computed CN counts for each signature in both clusters. To ensure reproducibility, we set a random seed (set.seed(123)).

For each CN signature, we compared the distributions of bootstrap replicates between the two clusters using the Anderson-Darling test (ad.test function from kSamples package). This non-parametric test was chosen for its sensitivity to differences in both the location and shape of distributions, making it suitable for comparing count data.

We calculated p-values for each CN signature and applied the Benjamini-Hochberg method to correct for multiple testing. Adjusted p-values < 0.05 were considered statistically significant. To quantify the magnitude of differences between clusters, we computed Cohen’s d effect size and its 95% confidence interval using the cohen.d function from the effsize package. Effect sizes were interpreted as follows: d < 0.2 (negligible), 0.2 ≤ d < 0.5 (small), 0.5 ≤ d < 0.8 (medium), and d ≥ 0.8 (large). We created histograms for each CN signature using ggplot2, displaying the distribution of bootstrap replicates for both clusters. These plots included vertical lines representing the original cluster counts, adjusted p-values from the bootstrap analysis, and Cohen’s d effect sizes. A combined plot showing all CN signatures was generated to provide an overview of the results.

By integrating bootstrap analysis with effect size calculations, this method provides a robust assessment of differences in CN signature distributions between clusters, considering both statistical significance and practical importance. We also performed this bootstrap analysis on single base substitution signatures with the same methodology.

### Substitution and VAF correlation analysis

To investigate the relationship between variant allele frequency and single-base substitutions across hallmark genes, we first preprocessed the dataset by filtering for regular single nucleotide substitutions. Variants involving insertions, deletions, or complex mutations were excluded to retain only substitutions where both the reference and mutated base were a single nucleotide (A, T, C, or G). We then categorized substitutions by gene and substitution type, grouping data accordingly. For each substitution type within hallmark genes, we calculated the frequency of occurrences and the mean VAF. To assess potential correlations between substitution frequency and VAF, we performed Pearson correlation analyses for each substitution type. We generated scatter plots to display the correlation between substitution frequency and mean VAF across substitution types.

## Statistics and data visualization

All statistical analyses were conducted using R statistical software (R v4.1.2), and p-values were adjusted for multiple comparisons where applicable using Benjamini Hochberg correction. All statistical analysis was performed using R. The ggplot2 (version 3.3.5) and ggpubr (version 0.4.0) packages were used for data visualization.

## Supplementary Information

Supplementary Information.

## Data Availability

All data used in this study are publicly available from TCGA (https://portal.gdc.cancer.gov/), it can be found here (https:/zenodo.org/records/15805088).
