# Supplementary material for: Distinct molecular profiles associated with early vs late genome instability in cancer
Source: Sci Rep. 2026 Apr 6;16:16468. doi: 10.1038/s41598-026-44528-y (PMC13216520; doi:10.1038/s41598-026-44528-y)
Supplement: Supplementary file 1 — Supplementary Information. [file 41598_2026_44528_MOESM1_ESM.docx]

Supplementary figures: Genetic and immune associations to clustering based on cancer hallmarks order

Supp Figure 1: Silent mutation rate

Supp Figure 2: Nonsilent mutation rate

Supp Figure 3: Summary table for Copy number signatures

| **CN** | **bootstrap_p_value** | **bootstrap_p_adjusted** | **effect_size** | **ci_lower** | **ci_upper** | **original_p_value** | **original_p_adjusted** |
| --- | --- | --- | --- | --- | --- | --- | --- |
| CN1 | 0 | 0 | 12.05884 | 11.67477 | 12.44292 | 1.12E-34 | 2.34E-33 |
| CN18 | 0 | 0 | -3.07241 | -3.2019 | -2.94292 | 3.46E-06 | 2.42E-05 |
| CN11 | 0 | 0 | -3.79425 | -3.94099 | -3.6475 | 7.31E-05 | 0.000307 |
| CN5 | 0 | 0 | -2.42169 | -2.53715 | -2.30623 | 0.042295 | 0.111025 |
| CN12 | 0 | 0 | -2.24998 | -2.36205 | -2.13791 | 0.123067 | 0.287157 |
| CN9 | 0 | 0 | -16.5239 | -17.0438 | -16.0041 | 0.16072 | 0.337512 |
| CN20 | 0 | 0 | -3.04939 | -3.17836 | -2.92042 | 0.178726 | 0.341203 |
| CN21 | 0 | 0 | -5.03044 | -5.2094 | -4.85149 | 0.300031 | 0.484666 |
| CN10 | 0 | 0 | -5.46634 | -5.65719 | -5.27549 | 0.651047 | 0.92699 |
| CN3 | 0 | 0 | -5.79166 | -5.99153 | -5.5918 | 0.843807 | 0.984441 |
| CN4 | 2.5E-241 | 4.7E-241 | -1.68102 | -1.78305 | -1.57899 | 1 | 1 |
| CN17 | 1.4E-231 | 2.5E-231 | 1.622015 | 1.520911 | 1.723119 | 8.46E-20 | 8.88E-19 |
| CN13 | 2.8E-161 | 4.6E-161 | -1.24061 | -1.33639 | -1.14484 | 0.026993 | 0.094477 |
| CN19 | 4.9E-119 | 7.3E-119 | -1.02078 | -1.11403 | -0.92754 | 1.53E-05 | 8.04E-05 |
| CN7 | 1.33E-33 | 1.86E-33 | 0.515457 | 0.426307 | 0.604607 | 0.843259 | 0.984441 |
| CN15 | 3.06E-31 | 4.02E-31 | -0.4857 | -0.57469 | -0.39671 | 0.742604 | 0.974668 |
| CN16 | 4.03E-27 | 4.98E-27 | 0.450306 | 0.361496 | 0.539116 | 0.984084 | 1 |
| CN14 | 1.69E-14 | 1.97E-14 | -0.32505 | -0.41333 | -0.23677 | 0.993402 | 1 |
| CN8 | 3.98E-08 | 4.4E-08 | 0.246035 | 0.157999 | 0.334072 | 0.662135 | 0.92699 |
| CN2 | 0.005406 | 0.005677 | -0.13066 | -0.21846 | -0.04286 | 0.03893 | 0.111025 |
| CN6 | 0.038106 | 0.038106 | -0.07525 | -0.16298 | 0.012489 | 0.236223 | 0.413391 |

Supp Figure 4: Summary table for Single Base Substitution signatures

| **signature** | **bootstrap_p_value** | **bootstrap_p_adjusted** | **effect_size** | **ci_lower** | **ci_upper** | **original_p_value** | **original_p_adjusted** |
| --- | --- | --- | --- | --- | --- | --- | --- |
| SBS12 | 0 | 0 | 1.364938 | 1.267554 | 1.462322 | 1 | 1 |
| SBS3 | 0 | 0 | 1.46255 | 1.363813 | 1.561287 | 3.67E-05 | 0.001065 |
| SBS30 | 0 | 0 | 1.4727 | 1.373818 | 1.571582 | 0.795373 | 1 |
| SBS52 | 0 | 0 | 1.385105 | 1.287448 | 1.482763 | 0.356762 | 0.820114 |
| SBS54 | 0 | 0 | 1.405397 | 1.307462 | 1.503333 | 0.178689 | 0.820114 |
| SBS7a | 0 | 0 | 1.422266 | 1.324096 | 1.520435 | 0.00063 | 0.007303 |
| SBS27 | 0 | 0 | 1.40015 | 1.302287 | 1.498013 | 0.480756 | 0.820114 |
| SBS53 | 8.4E-113 | 6.1E-112 | 1.136544 | 1.042023 | 1.231066 | 0.480756 | 0.820114 |
| SBS5 | 6E-110 | 3.8E-109 | 0.976677 | 0.88389 | 1.069464 | 0.178726 | 0.820114 |
| SBS59 | 2.6E-99 | 1.53E-98 | 0.836788 | 0.745325 | 0.928251 | 0.688733 | 0.894189 |
| SBS37 | 1.88E-97 | 9.92E-97 | 0.846311 | 0.754763 | 0.937858 | 1 | 1 |
| SBS6 | 9.17E-97 | 4.43E-96 | 0.888956 | 0.797021 | 0.980891 | 0.255041 | 0.820114 |
| SBS22 | 2.84E-93 | 1.27E-92 | 0.792875 | 0.701788 | 0.883961 | 0.688395 | 0.894189 |
| SBS49 | 2.16E-91 | 8.93E-91 | 0.857953 | 0.766301 | 0.949604 | 0.480756 | 0.820114 |
| SBS28 | 5.74E-85 | 2.22E-84 | 0.64625 | 0.556284 | 0.736215 | 0.231085 | 0.820114 |
| SBS29 | 1.82E-80 | 6.58E-80 | 0.708947 | 0.618528 | 0.799365 | 0.633448 | 0.894189 |
| SBS2 | 7.79E-75 | 2.66E-74 | 0.617042 | 0.527274 | 0.70681 | 0.00029 | 0.004223 |
| SBS8 | 5.36E-60 | 1.73E-59 | -0.68089 | -0.7711 | -0.59068 | 1 | 1 |
| SBS7b | 3.33E-56 | 1.02E-55 | 0.661003 | 0.570934 | 0.751072 | 0.573539 | 0.894189 |
| SBS11 | 9.26E-48 | 2.68E-47 | 0.157509 | 0.069667 | 0.24535 | 0.480756 | 0.820114 |
| SBS32 | 1.78E-47 | 4.92E-47 | -0.67751 | -0.7677 | -0.58732 | 0.275156 | 0.820114 |
| SBS7c | 5.53E-44 | 1.46E-43 | 0.194223 | 0.106311 | 0.282135 | 0.356762 | 0.820114 |
| SBS18 | 1.54E-42 | 3.88E-42 | -0.50968 | -0.5988 | -0.42056 | 0.214277 | 0.820114 |
| SBS41 | 2.51E-41 | 6.07E-41 | -0.50816 | -0.59727 | -0.41905 | 1 | 1 |
| SBS7d | 9.2E-41 | 2.13E-40 | 0.13901 | 0.051199 | 0.226821 | 0.060858 | 0.588292 |
| SBS10a | 2.37E-40 | 5.28E-40 | -0.09848 | -0.18624 | -0.01072 | 1 | 1 |
| SBS23 | 3.55E-40 | 7.63E-40 | 0.202644 | 0.114714 | 0.290574 | 1 | 1 |
| SBS10b | 3.69E-40 | 7.64E-40 | 0.262776 | 0.174693 | 0.350859 | 0.452896 | 0.820114 |
| SBS39 | 7.26E-40 | 1.45E-39 | 0.39494 | 0.306383 | 0.483496 | 4.58E-06 | 0.000266 |
| SBS58 | 1.4E-38 | 2.71E-38 | 0.31793 | 0.229673 | 0.406188 | 0.68339 | 0.894189 |
| SBS46 | 3.99E-38 | 7.47E-38 | -0.24656 | -0.3346 | -0.15852 | 0.866465 | 1 |
| SBS48 | 1.25E-37 | 2.27E-37 | 0.154942 | 0.067105 | 0.242779 | 0.480756 | 0.820114 |
| SBS38 | 2.52E-37 | 4.43E-37 | 0.341643 | 0.2533 | 0.429986 | 1 | 1 |
| SBS19 | 8.82E-37 | 1.5E-36 | 0.290508 | 0.202342 | 0.378675 | 0.072257 | 0.598701 |
| SBS31 | 2E-36 | 3.31E-36 | -0.3095 | -0.39773 | -0.22127 | 0.688395 | 0.894189 |
| SBS4 | 3.2E-36 | 5.15E-36 | -0.14845 | -0.23628 | -0.06063 | 0.376322 | 0.820114 |
| SBS1 | 5.6E-35 | 8.78E-35 | -0.1391 | -0.22691 | -0.05129 | 0.285382 | 0.820114 |
| SBS36 | 2.06E-34 | 3.15E-34 | 0.179768 | 0.091886 | 0.26765 | 0.53553 | 0.862799 |
| SBS16 | 1.05E-33 | 1.55E-33 | 0.084284 | -0.00346 | 0.172029 | 0.372699 | 0.820114 |
| SBS33 | 1.13E-33 | 1.64E-33 | 0.060784 | -0.02694 | 0.14851 | 0.272093 | 0.820114 |
| SBS20 | 1.27E-33 | 1.79E-33 | 0.314119 | 0.225874 | 0.402363 | 0.672491 | 0.894189 |
| SBS50 | 1.36E-33 | 1.88E-33 | -0.02606 | -0.11377 | 0.061653 | 0.431355 | 0.820114 |
| SBS26 | 1.47E-33 | 1.98E-33 | -0.21623 | -0.30419 | -0.12827 | 0.12166 | 0.820114 |
| SBS60 | 2.13E-33 | 2.81E-33 | -0.11507 | -0.20284 | -0.02729 | 0.480756 | 0.820114 |
| SBS14 | 2.65E-33 | 3.41E-33 | -0.15887 | -0.24671 | -0.07103 | 1 | 1 |
| SBS44 | 4.11E-33 | 5.19E-33 | 0.033402 | -0.05431 | 0.121113 | 0.850402 | 1 |
| SBS35 | 1.13E-32 | 1.39E-32 | 0.093061 | 0.005308 | 0.180814 | 0.529973 | 0.862799 |
| SBS51 | 6.04E-32 | 7.3E-32 | 0.134797 | 0.046992 | 0.222602 | 0.15947 | 0.820114 |
| SBS21 | 2.7E-31 | 3.2E-31 | 0.264221 | 0.176134 | 0.352308 | 0.211138 | 0.820114 |
| SBS25 | 2.89E-31 | 3.35E-31 | 0.176402 | 0.088526 | 0.264278 | 0.387444 | 0.820114 |
| SBS17a | 5.38E-31 | 6.12E-31 | -0.00453 | -0.09224 | 0.083175 | 1 | 1 |
| SBS15 | 3.16E-30 | 3.53E-30 | -0.07945 | -0.16719 | 0.008289 | 0.328756 | 0.820114 |
| SBS57 | 5.26E-29 | 5.76E-29 | -0.1211 | -0.20889 | -0.03332 | 0.873647 | 1 |
| SBS42 | 1.01E-28 | 1.08E-28 | -0.12626 | -0.21405 | -0.03847 | 0.000291 | 0.004223 |
| SBS17b | 0.026608 | 0.028059 | -0.09101 | -0.17876 | -0.00326 | 0.157799 | 0.820114 |
| SBS13 | 0.76513 | 0.792456 | 0.004989 | -0.08272 | 0.092694 | 0.289741 | 0.820114 |
| SBS24 | 0.99999 | 0.99999 | 0.009825 | -0.07788 | 0.097531 | 0.693768 | 0.894189 |
| SBS40 | 0.99949 | 0.99999 | 0.006074 | -0.08163 | 0.09378 | 0.644583 | 0.894189 |

Supp Figure 5: Correlation between the number of specific substitutions and the mean VAF of cancer hallmark genes
